# Robust Sequential Fusion Estimation Based on Adaptive Innovation Event-Triggered Mechanism for Uncertain Networked Systems

**DOI:** 10.1155/2022/8228525

**Published:** 2022-11-30

**Authors:** Ke Xu, Xinye Li, Jie Wang, Yuan Gao

**Affiliations:** School of Electronic Engineering, Heilongjiang University, Harbin 150080, China

## Abstract

In order to reduce the transmission pressure of the networked system and improve its robust performance, an adaptive innovation event-triggered mechanism is designed for the first time, and based on this mechanism, the robust local filtering algorithm for the multi-sensor networked system with uncertain noise variances and correlated noises is presented. To avoid calculating the complex error cross-covariance matrices, applying the sequential fusion idea, the robust sequential covariance intersection (SCI) and sequential inverse covariance intersection (SICI) fusion estimation algorithms are proposed, and their robustness is analyzed. Finally, it is verified in the simulation example that the proposed adaptive innovation event-triggered mechanism can reduce the communication burden, the robust local filtering algorithm is effective for the uncertainty generated by the unknown noise variances, and two robust sequential fusion estimators show good robustness, respectively.

## 1. Introduction

It is well known that Kalman filtering algorithm is the most widely used filtering method at present. However, the traditional Kalman filter requires precisely known model parameters and noise variances, and the above requirements cannot be easily satisfied in practical applications. When the sensor system is in the stochastic uncertain state, the traditional Kalman filter will decrease the accuracy or even diverge [1], which can be improved by designing a robust Kalman filtering algorithm to cope with all allowable uncertainties and obtain the minimum upper bound on the actual filtering error variance [2]. The robust Kalman estimator was designed for the sensor systems with mixed uncertainties by converting the original system to a new system with uncertain noise variances only by the fictitious noise technique in [3].

Kalman filtering algorithm was first applied in single sensor systems, but with the rapid development of the network communication technology, more and more sensors are widely used in military, aerospace, transportation, and other fields [4], and the data of each node (sensor) are transmitted through communication channels, which form the sensor networked system. Multi-sensor information fusion techniques, fusing and processing information from multiple nodes, have been developed to obtain more accurate data. Currently, the basic fusion methods in the field of state estimation are centralized fusion and distributed fusion methods. The centralized fusion method uses all information to obtain the global optimal estimation. But in actual applications, a large amount of data should be handled, so the computational burden of the centralized fusion method is high and its fault tolerance and reliability are poor. The distributed fusion method fused the local state estimation transmitted from each local sensor to the fusion center, whose mainstream methods include the distributed fusion algorithms weighted by matrices [5], diagonal matrices [6], and scalars [7]. Those algorithms have lower accuracy but a less computational burden and a parallel structure with higher fault tolerance.

Many results have been achieved in the research on the networked systems with uncertainties. In [8–10], the local and fused robust time-varying Kalman predictors, filters, and smoothers were proposed for uncertain systems with uncertain noise variances. The robust Kalman estimators were designed for sensor systems with mixed uncertainties in [11], which was achieved by converting the original system into a new system with uncertain noise variances and applying the augmented state, derandomization, and fictitious noise technologies.

In the networked systems, some factors, such as the discretization of the continuous system [12, 13], the normalization of the generalized system [14], the environment involving the same noise source, and so on, will lead to the correlation between the process noise and the observation noise from the single sensor or that between the observation noises from two different sensors. In [15], an optimal sequential filtering algorithm was proposed for linear time-varying discrete systems, where the observation noises are cross-correlated. In [16], a distributed fusion filter was presented for the multi-sensor system with finite-step autocorrelation of process noises and cross-correlation among observation noises, using the matrix-weighted fusion estimation algorithm.

Some research works focus on the sensor networked system with both uncertainties and correlated noises. A distributed weighted fusion robust Kalman filtering algorithm was proposed for uncertain networked systems with one-step autocorrelated and two-step cross-correlated noises in [17]. In [18], a robust extended Kalman filter was proposed for nonlinear discrete-time systems with unknown inputs and correlated noises.

The above-used fusion methods require known estimation error cross-covariances among the local sensors exactly, which are not easily available in practice, while covariance intersection (CI) fusion algorithm can overcome this weakness and reduce the computational burden significantly [19]. Inverse covariance intersection (ICI) fusion algorithm was proposed in [20, 21], which was proved to be less conservative than CI fusion algorithm and has tight consistency. To extend the above two fusion algorithms to the multi-sensor systems and reduce the computational burden and the complexity of the batch algorithm [22, 23], based on the sequential fusion idea [24], the sequential inverse covariance intersection (SICI) fusion algorithm was proposed in [25, 26], which is equivalent to the multiple two-sensor ICI fusion estimation, inherits the advantages of ICI fusion algorithm, and is more suitable for the actual applications.

The sampling period is usually set in the traditional multi-sensor networked systems, which means that the time-triggered mechanism is used for data transmission. But due to the limited bandwidth, the transmission process may generate packet loss, while using an event-triggered mechanism can reduce the transmission pressure of the networked system and save the cost of communication resources. So, more researchers are concerned with the study of the event-trigger mechanisms. For example, the mode-dependent event-triggered mechanism was designed for the discrete-time fuzzy Markov jump singularly perturbed systems in [27], and the adaptive event-triggered mechanism was designed for the semi-Markovian switching cyber-physical systems in [28]. Based on the innovation triggering mechanism [29], an optimal state estimation algorithm for a multi-sensor system with correlated noises was presented by an iterative white-noise estimator, which can significantly reduce the communication requirements compared with the traditional time-triggered scheme, although the estimation performance is slightly degraded. In [30], based on an innovation event-triggered mechanism, a distributed fusion estimation algorithm was proposed for the multi-sensor nonlinear networked system with stochastic transmission delays, which reduced the measurement transmission data from each communication channel and ensured the estimation performance. Under the linear minimum variance criterion, based on the innovation event-triggered mechanism, an event-triggered optimal sequential fusion filter for the multi-sensor correlated-noise systems was proposed in [31] to reduce the communication rate from the local sensors to the fusion center. However, none of those mentioned research studies consider the situation that the networked system has both uncertainties and limited communication resources simultaneously.

In order to reduce the network transmission pressure and the computational burden on the fusion center and to improve the robustness of the estimators, an adaptive innovation event-triggered mechanism is presented, and for the multi-sensor networked systems with unknown noise variances and correlated noises, based on the arriving order of the local robust estimators at the fusion center, the sequential covariance intersection and sequential inverse covariance intersection fusion robust estimation algorithms are presented. The above sequential fusion algorithms can avoid the calculation of the estimation error cross-covariance matrices, solve the networked uncertainties brought by the unknown actual noise variances and actual error cross-covariances, and reduce the risk of data transmission failure. They have good stability and robustness and are more suitable for real-time application.

## 2. Problem Description

### 2.1. System Model

Considering the linear discrete time-varying multi-sensor networked systems with uncertain noise variances and correlated noises,

(1) $$\begin{array}{c} x\left(k+1\right)=\Phi \left(k\right)x\left(k\right)+w\left(k\right)\text{,} \end{array}$$

(2) $$\begin{array}{c} y_{i}\left(k\right)=H_{i}\left(k\right)x\left(k\right)+v_{i}\left(k\right)\text{,} \end{array}$$

(3) $$\begin{array}{c} v_{i}\left(k\right)=\eta_{i}\left(k\right)+\beta_{i}\left(k\right)w\left(k-1\right),i=1,\cdots ,L\text{,} \end{array}$$

where *x*(*k*)*∈R*^*n*^ is the state, *w*(*k*)*∈R*^*r*^ is the process noise, *y*_*i*_(*k*)*∈R*^*m*_*i*_^ is the observation of the *i*th sensor, *v*_*i*_(*k*)*∈R*^*m*_*i*_^ is the observation noise of the *i*th sensor, and *η*_*i*_(*k*)*∈R*^*m*_*i*_^ is the white noise uncorrelated with *w*(*k*). Φ(*k*), *H*_*i*_(*k*), and *β*_*i*_(*k*) are the time-varying matrices with *n×n*, *m*_*i*_*×n*, and *m*_*i*_*×r* dimensions, respectively, and *L* is the number of sensors.

**Assumption 1 .**

*w*(*k*) and *η*_*i*_(*k*) are uncorrelated white noises with zero mean and uncertain actual variance matrices $\overset{¯}{Q}\left(k\right)$ and ${\overset{¯}{\sigma }}_{\eta_{i}}^{2}\left(k\right)$, respectively, whose conservative upper bounds *Q*, *σ*_*η*_*i*__^2^(*k*) are known and satisfy the relations

(4) $$\begin{array}{c} \overset{¯}{Q}\left(k\right)\leq Q\left(k\right),{\overset{¯}{\sigma }}_{i}\left(k\right)\leq \sigma_{i}\left(k\right),i=1,\cdots ,L,\forall k\text{.} \end{array}$$

From (3) and Assumption 1, *v*_*i*_(*k*) is a white noise with zero mean, uncertain actual variance matrix ${\overset{¯}{R}}_{ij}\left(k\right)={\overset{¯}{\sigma }}_{\eta_{i}}^{2}\left(k\right)\delta_{ij}+\beta_{i}\left(k\right)\overset{¯}{Q}\left(k-1\right)\beta_{j}^{T}\left(k\right)$, and conservative variance matrix upper bound *R*_*ij*_*=σ*_*η*_*i*__^2^(*k*)*δ*_*ij*_*+β*_*i*_(*k*)*Q*(*k−*1)*β*_*j*_^T^(*k*), which satisfies the relation

(5) $$\begin{array}{c} {\overset{¯}{R}}_{ij}\left(k\right)\leq R_{ij}\left(k\right),i=1,\cdots ,L,\forall k\text{.} \end{array}$$

The uncertain actual one-step correlation matrix between *w*(*k*) and *v*_*i*_(*k*) is ${\overset{¯}{S}}_{i}\left(k\right)=\overset{¯}{Q}\left(k-1\right)\beta_{i}^{T}\left(k\right)$, and it yields

(6) $$\begin{array}{c} E\left\{\left[\begin{array}{c} w\left(k-1\right)\\ v_{i}\left(k\right) \end{array}\right]\left[\begin{array}{cc} w^{T}\left(t-1\right) & v_{j}^{T}\left(t\right) \end{array}\right]\right\}=\left[\begin{array}{cc} \overset{¯}{Q}\left(k-1\right) & {\overset{¯}{S}}_{j}\left(k\right)\\ {\overset{¯}{S}}_{i}^{T}\left(k\right) & {\overset{¯}{R}}_{ij}\left(k\right) \end{array}\right]\delta_{kt}\text{,} \end{array}$$

where *δ*_*kk*_*=*1, *δ*_*kt*_*=*0(*t≠k*).

**Assumption 2 .**

Suppose that the state *x*(0) has the mean *μ*_0_ and the uncertain actual variance matrix $\overset{¯}{P}\left(0|0\right)$ and is uncorrelated with *w*(*k*) and *v*_*i*_(*k*), i.e.,

(7) $$\begin{array}{c} E\left[x\left(0\right)\right]=\mu_{0},E\left[\left(x\left(0\right)-\mu_{0}\right){\left(x\left(0\right)-\mu_{0}\right)}^{T}\right]=P_{0}\text{,}\\ E\left[x\left(0\right)w^{T}\left(k\right)\right]=0,E\left[x\left(0\right)v_{i}^{T}\left(k\right)\right]=0,\forall k\text{,} \end{array}$$

where exists the relation

(8) $$\begin{array}{c} {\overset{¯}{P}}_{i}\left(0|0\right)\leq P_{i}\left(0|0\right)\text{,} \end{array}$$

where *P*_*i*_(0*|*0) is the known conservative upper bound of ${\overset{¯}{P}}_{i}\left(0|0\right)$.

### 2.2. Adaptive Innovation Event-Triggered Mechanism

In order to reduce the pressure of network communication transmission, an adaptive innovation event-triggered mechanism is designed, introducing Bernoulli random variable *γ*_*i*_(*k*) to describe the transmission state of the *i*th sensor and defining the innovation *ε*_*i*_(*k*) as the difference between the observation and its estimation, i.e., εik=yik−y^ik|k−1. When the product *ε*_*i*_^*T*^(*k*)*ε*_*i*_(*k*) of the *i*th sensor exceeds the threshold value *θ*_*i*_, the current observation will be transmitted; otherwise, no transmission will be performed. It can be described as

(9) $$\begin{array}{c} \gamma_{i}\left(k\right)=\left\{\begin{array}{cc} 1\text{,} & \varepsilon_{i}^{T}\left(k\right)\varepsilon_{i}\left(k\right)>\theta_{i}\left(k\right)\text{,}\\ 0\text{,} & otherwise\text{,} \end{array}\right. \end{array}$$

with the threshold value

(10) $$\begin{array}{c} \theta_{i}\left(k\right)=ae^{-b+c{\left\|\varepsilon_{i}\left(l_{i}\left(k\right)\right)\right\|}_{2}}\text{,} \end{array}$$

where *a*,*b*,*c≥*0 are the appropriate parameters and *l*_*i*_(*k*) denotes the last triggered moment, updated with time

(11) $$\begin{array}{c} l_{i}\left(k\right)=\left\{\begin{array}{c} k\text{,}\\ l_{i}\left(k-1\right)\text{,} \end{array}\right.\;\begin{array}{c} \gamma_{i}\left(k\right)=1\text{,}\\ \gamma_{i}\left(k\right)=0\text{.} \end{array} \end{array}$$

**Figure 1:**
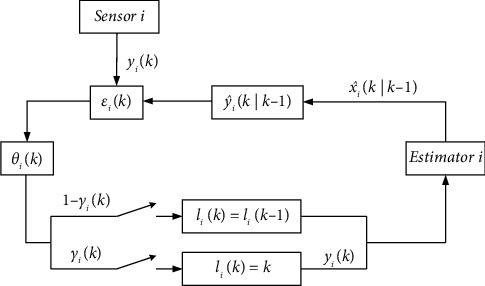
Adaptive innovation event-triggered mechanism structure diagram.

If the product *ε*_*i*_^T^(*k*)*ε*_*i*_(*k*) does not exceed the threshold value of the last transmission moment, the threshold value will remain unchanged; otherwise, since the threshold value is a monotonically decreasing function on [0,*+∞*), the threshold value will decrease with the increase of the innovation, and thus it will perform the adaptive regulation. When *a=*0, the adaptive innovation event-triggered mechanism will degenerate to the conventional time-triggered mechanism, and when *c=*0, it will deteriorate to the event-triggered mechanism with a fixed threshold value. The structure diagram of the adaptive innovation event-triggered mechanism is shown in Figure 1.

This paper aims to propose the robust SCI and SICI fusion estimation algorithms for multi-sensor networked systems with uncertain noise variances and correlated noises, based on the adaptive innovation event-triggered mechanism.

## 3. Robust Local Filtering Algorithm

For a multi-sensor networked system with known conservative upper bounds of noise variances and correlated noises, based on the adaptive innovation event-triggered mechanism, the classical Kalman filtering theory is applied to the following lemma.

**Lemma 1 .**

(see [32]). Under Assumptions 1 and 2, based on the adaptive innovation event-triggered mechanism, the local conservative recursive Kalman filter for the uncertain networked systems (1)–(3) is

(12) x^ik|k=x^ik|k−1+γikKikεik,Pik|k=Pik|k−1−γikKikQεikKiTk,εik=yik−Hikx^ik|k−1,Qεik=HikPik|k−1HiTk+HikSik+SiTkHiTk+Riik,Kik=Pik|k−1HiTk+SikQεi−1k,x^ik+1|k=Φkx^ik|k,Pik+1|k=ΦkPik|kΦTk+Qk,x^i0|−1=μ0,Pi0|−1=P0,

with one-step prediction error variance *P*_*i*_(*k+*1*|k*) which satisfies the Riccati equation

(13) $$\begin{array}{c} P_{i}\left(k+1|k\right)=\Phi \left(k\right)P_{i}\left(k|k-1\right)\Phi^{T}\left(k\right)-\left[\Phi \left(k\right)P_{i}\left(k|k-1\right)H_{i}^{T}\left(k\right)+\Phi \left(k\right)S_{i}\left(k\right)\right]{\left[H_{i}\left(k\right)P_{i}\left(k|k-1\right)H_{i}^{T}\left(k\right)+H_{i}\left(k\right)S_{i}\left(k\right)+S_{i}^{T}\left(k\right)H_{i}^{T}\left(k\right)+R_{ii}\left(k\right)\right]}^{-1}\times {\left[\Phi \left(k\right)P_{i}\left(k|k-1\right)H_{i}^{T}\left(k\right)+\Phi \left(k\right)S_{i}\left(k\right)\right]}^{T}+Q\left(k\right) \end{array}$$

When the local sensor does not meet the threshold condition at some moment, the estimator cannot receive the observation, and then the estimator will perform a prediction at that moment.

**Remark 1 .**

The conservative observations are unknown and not available, since they can only be theoretically generated by the worst-case systems with known conservative upper bounds *Q*(*k*), *R*_*ii*_(*k*), and *S*_*i*_(*k*) of noise variances, while the actual observations generated by the actual system (2) with actual unknown noise variances $\overset{¯}{Q}\left(k\right)$, ${\overset{¯}{R}}_{ii}\left(k\right)$, and ${\overset{¯}{S}}_{i}\left(k\right)$ are available. Therefore, the local Kalman filter only can be obtained using the actual observations in Lemma 1, which is called the conservative Kalman filter.

Defining the augmented noise *λ*_*i*_(*k*)*=*[*w*^T^(*k−*1),*v*_*i*_^T^(*k*)]^T^, the conservative and actual covariance matrices between *λ*_*i*_(*k*) and *λ*_*j*_(*k*) are

(14) $$\begin{array}{c} \Lambda_{ij}\left(k\right)=E\left[\lambda_{i}\left(k\right)\lambda_{j}^{T}\left(k\right)\right]=\left[\begin{array}{cc} Q\left(k-1\right) & S_{j}\left(k\right)\\ S_{i}^{T}\left(k\right) & R_{ij} \end{array}\right]=\left[\begin{array}{cc} Q\left(k-1\right) & Q\left(k-1\right)\beta_{j}^{T}\left(k\right)\\ \beta_{i}\left(k\right)Q\left(k-1\right) & \beta_{i}\left(k\right)Q\left(k-1\right)\beta_{j}^{T}\left(k\right)+\sigma_{\eta_{i}}^{2}\left(k\right)\delta_{ij} \end{array}\right]\text{,} \end{array}$$

(15) $$\begin{array}{c} {\overset{¯}{\Lambda }}_{ij}\left(k\right)=\left[\begin{array}{cc} \overset{¯}{Q}\left(k-1\right) & {\overset{¯}{S}}_{j}\left(k\right)\\ {\overset{¯}{S}}_{i}^{T}\left(k\right) & {\overset{¯}{R}}_{ij} \end{array}\right]=\left[\begin{array}{cc} \overset{¯}{Q}\left(k-1\right) & \overset{¯}{Q}\left(k-1\right)\beta_{j}^{T}\left(k\right)\\ \beta_{i}\left(k\right)\overset{¯}{Q}\left(k-1\right) & \beta_{i}\left(k\right)\overset{¯}{Q}\left(k-1\right)\beta_{j}^{T}\left(k\right)+{\overset{¯}{\sigma }}_{\eta_{i}}^{2}\left(k\right)\delta_{ij} \end{array}\right]\text{.} \end{array}$$

The local filtering errors can be derived as

(16) x˜ik|k=xk−x^ik|k=Ψfikx˜ik−1|k−1+Γikλik,

where

(17) $$\begin{array}{c} \Psi_{fi}\left(k\right)=\left[I_{n}-\gamma_{i}\left(k\right)K_{i}\left(k\right)H_{i}\left(k\right)\right]\Phi \left(k-1\right)\text{,}\\ \Gamma_{i}^{\gamma_{i}}\left(k\right)=\left[I_{n}-\gamma_{i}\left(k\right)K_{i}\left(k\right)H_{i}\left(k\right),-\gamma_{i}\left(k\right)K_{i}\left(k\right)\right]\text{.} \end{array}$$

From (16), according to Assumptions 1 and 2 and the projection theory, it is easily known that *w*(*k−*1), *v*_*i*_(*k*) and ${\overset{˜}{x}}_{i}\left(k-1|k-1\right)$ are not correlated.

When *γ*_*i*_(*k*)*=*1, the local conservative filtering error variance matrix $P_{i}\left(k|k\right)=E\left[{\overset{˜}{x}}_{i}\left(k|k\right){\overset{˜}{x}}_{i}^{T}\left(k|k\right)\right]$ satisfies the Lyapunov equation

(18) $$\begin{array}{c} P_{i}\left(k|k\right)=\Psi_{fi}^{1}\left(k\right)P_{i}\left(k-1|k-1\right){\left(\Psi_{fi}^{1}\left(k\right)\right)}^{T}+\Gamma_{i}^{1}\left(k\right)\Lambda_{ii}\left(k\right){\left(\Gamma_{i}^{1}\left(k\right)\right)}^{T}\text{,} \end{array}$$

where

(19) $$\begin{array}{c} \Psi_{fi}^{1}\left(k\right)=\left[I_{n}-K_{i}\left(k\right)H_{i}\left(k\right)\right]\Phi \left(k-1\right)\text{,}\\ \Gamma_{i}^{1}\left(k\right)=\left[I_{n}-K_{i}\left(k\right)H_{i}\left(k\right),-K_{i}\left(k\right)\right]\text{.} \end{array}$$

When *γ*_*i*_(*k*)*=*0, the local conservative filtering error variance matrix *P*_*i*_(*k|k*) satisfies the Lyapunov equation

(20) $$\begin{array}{c} P_{i}\left(k|k\right)=\Psi_{fi}^{0}\left(k\right)P_{i}\left(k-1|k-1\right){\left(\Psi_{fi}^{0}\left(k\right)\right)}^{T}+\Gamma_{i}^{0}\left(k\right)\Lambda_{ii}\left(k\right){\left(\Gamma_{i}^{0}\left(k\right)\right)}^{T}\text{,} \end{array}$$

where

(21) $$\begin{array}{c} \Psi_{fi}^{0}\left(k\right)=\Phi \left(k-1\right)\text{,}\\ \Gamma_{i}^{0}\left(k\right)=\left[\begin{array}{cc} I_{n}\text{,} & 0 \end{array}\right]\text{.} \end{array}$$

To sum up, the local conservative filtering error variance matrix *P*_*i*_(*k|k*) satisfies the universal Lyapunov equation

(22) $$\begin{array}{c} P_{i}\left(k|k\right)=\Psi_{fi}\left(k\right)P_{i}\left(k-1|k-1\right)\Psi_{fi}^{T}\left(k\right)+\Gamma_{i}^{\gamma_{i}}\left(k\right)\Lambda_{ii}\left(k\right){\left(\Gamma_{i}^{\gamma_{i}}\left(k\right)\right)}^{T}\text{,} \end{array}$$

with the initial value *P*_*i*_(0*|*0)*=P*_0_.

Similarly, according to Assumption 1, the actual local filtering error variance matrix ${\overset{¯}{P}}_{i}\left(k|k\right)$ satisfies the Lyapunov equation

(23) $$\begin{array}{c} {\overset{¯}{P}}_{i}\left(k|k\right)=\Psi_{fi}\left(k\right){\overset{¯}{P}}_{i}\left(k-1|k-1\right)\Psi_{fi}^{T}\left(k\right)+\Gamma_{i}^{\gamma_{i}}\left(k\right){\overset{¯}{\Lambda }}_{ii}\left(k\right){\left(\Gamma_{i}^{\gamma_{i}}\left(k\right)\right)}^{T}\text{,} \end{array}$$

with the initial value ${\overset{¯}{P}}_{i}\left(0|0\right)=P_{0}$.

When *γ*_*i*_(*k*)*=*1 and *γ*_*j*_(*k*)*=*1, the actual local filtering error cross-covariance matrix ${\overset{¯}{P}}_{ij}\left(k|k\right)=E\left[{\overset{˜}{x}}_{i}\left(k|k\right){\overset{˜}{x}}_{j}^{T}\left(k|k\right)\right]$ is

(24) $$\begin{array}{c} {\overset{¯}{P}}_{ij}\left(k|k\right)=\Psi_{fi}^{1}\left(k\right){\overset{¯}{P}}_{ij}\left(k-1|k-1\right){\left(\Psi_{fj}^{1}\left(k\right)\right)}^{T}+\Gamma_{i}^{1}\left(k\right){\overset{¯}{\Lambda }}_{ij}\left(k\right){\left(\Gamma_{j}^{1}\left(k\right)\right)}^{T}\text{.} \end{array}$$

When *γ*_*i*_(*k*)*=*1 and *γ*_*j*_(*k*)*=*0, the actual local filtering error cross-covariance matrix ${\overset{¯}{P}}_{ij}\left(k|k\right)$ is

(25) $$\begin{array}{c} {\overset{¯}{P}}_{ij}\left(k|k\right)=\Psi_{fi}^{1}\left(k\right){\overset{¯}{P}}_{ij}\left(k-1|k-1\right){\left(\Psi_{fj}^{0}\left(k\right)\right)}^{T}+\Gamma_{i}^{1}\left(k\right){\overset{¯}{\Lambda }}_{ij}\left(k\right){\left(\Gamma_{j}^{0}\left(k\right)\right)}^{T}\text{.} \end{array}$$

When *γ*_*i*_(*k*)*=*0 and *γ*_*j*_(*k*)*=*1, the actual local filtering errors cross-covariance matrix ${\overset{¯}{P}}_{ij}\left(k|k\right)$ is

(26) $$\begin{array}{c} {\overset{¯}{P}}_{ij}\left(k|k\right)=\Psi_{fi}^{0}\left(k\right){\overset{¯}{P}}_{ij}\left(k-1|k-1\right){\left(\Psi_{fj}^{1}\left(k\right)\right)}^{T}+\Gamma_{i}^{0}\left(k\right){\overset{¯}{\Lambda }}_{ij}\left(k\right){\left(\Gamma_{j}^{1}\left(k\right)\right)}^{T}\text{.} \end{array}$$

When *γ*_*i*_(*k*)*=*0 and *γ*_*j*_(*k*)*=*0, the actual local filtering error cross-covariance matrix ${\overset{¯}{P}}_{ij}\left(k|k\right)$ is

(27) $$\begin{array}{c} {\overset{¯}{P}}_{ij}\left(k\left|k\right.\right)=\Psi_{fi}^{0}\left(k\right){\overset{¯}{P}}_{ij}\left(k-1\left|k-1\right.\right){\left(\Psi_{fi}^{0}\left(k\right)\right)}^{T}+\Gamma_{i}^{0}\left(k\right)\Lambda_{ii}\left(k\right){\left(\Gamma_{j}^{0}\left(k\right)\right)}^{T}\text{.} \end{array}$$

In a word, the actual local filtering error cross-covariance matrix ${\overset{¯}{P}}_{ij}\left(k\left|k\right.\right)$ can be universally described as

(28) $$\begin{array}{c} P_{ij}\left(k\left|k\right.\right)=\Psi_{fi}\left(k\right){\overset{¯}{P}}_{ij}\left(k-1\left|k-1\right.\right)\Psi_{fj}^{T}\left(k\right)+\Gamma_{i}^{\gamma_{i}}\left(k\right){\overset{¯}{\Lambda }}_{ij}\left(k\right){\left(\Gamma_{j}^{\gamma_{i}}\left(k\right)\right)}^{T}\text{.} \end{array}$$

**Lemma 2 (see [9]).**

If a matrix Λ*∈R*^*r×r*^ is a semi-positive definite matrix, i.e., Λ*≥*0, a matrix Λ_*δ*_*∈R*^*rL×rL*^ is also a semi-positive definite matrix with form as

(29) $$\begin{array}{c} \Lambda_{\delta }={\left[\begin{array}{ccc} \Lambda & \cdots & \Lambda \\ \vdots & \ddots & \vdots \\ \Lambda & \cdots & \Lambda \end{array}\right]}_{rL\times rL}\geq 0\text{.} \end{array}$$

**Lemma 3 (see [9]).**

If the matrix *R*_*i*_*∈R*^*m*_*i*_*×m*_*i*_^ is a semi-positive definite matrix, i.e., *R*_*i*_*≥*0, the diagonal matrix *R*_*δ*_*=di* *ag*(*R*_1_,*⋯*,*R*_*L*_)*∈R*^*m×m*^ is also semi-positive definite, i.e.,

(30) $$\begin{array}{c} R_{\delta }=diag\left(R_{1},\cdots ,R_{L}\right)\geq 0\text{,} \end{array}$$

where *m=m*_1_*+⋯+m*_*L*_ and di ag(*·*) denotes the block diagonal matrix.

**Theorem 1 .**

Under Assumptions 1 and 2, for the uncertain networked systems (1)–(3) with known conservative upper bounds and correlated noises, based on the adaptive innovation event-triggered mechanism, the local conservative Kalman filter x^ik|k is robust, that is, for all admissible uncertain $\overset{¯}{Q}\left(k\right)$, ${\overset{¯}{R}}_{ii}\left(k\right)$, and ${\overset{¯}{P}}_{i}\left(0|0\right)$ satisfying (4), it has

(31) $$\begin{array}{c} {\overset{¯}{P}}_{i}\left(k|k\right)\leq P_{i}\left(k|k\right)\text{,} \end{array}$$

where *P*_*i*_(*k|k*) is the minimal upper bound of ${\overset{¯}{P}}_{i}\left(k|k\right)$. This local conservative Kalman filter is called the robust local event-triggered Kalman filter.

**Proof**

According to Assumption 1, (14), and (15), there are

(32) $$\begin{array}{c} \Lambda_{ii}\left(k\right)=\left[\begin{array}{cc} I_{n} & 0\\ 0 & \beta_{i}\left(k\right) \end{array}\right]\left[\begin{array}{cc} Q\left(k-1\right) & Q\left(k-1\right)\\ Q\left(k-1\right) & Q\left(k-1\right) \end{array}\right]\times {\left[\begin{array}{cc} I_{n} & 0\\ 0 & \beta_{i}\left(k\right) \end{array}\right]}^{T}+\left[\begin{array}{cc} 0 & 0\\ 0 & \sigma_{\eta_{i}}^{2}\left(k\right) \end{array}\right]\text{,}\\ {\overset{¯}{\Lambda }}_{ii}\left(k\right)=\left[\begin{array}{cc} I_{n} & 0\\ 0 & \beta_{i}\left(k\right) \end{array}\right]\left[\begin{array}{cc} \overset{¯}{Q}\left(k-1\right) & \overset{¯}{Q}\left(k-1\right)\\ \overset{¯}{Q}\left(k-1\right) & \overset{¯}{Q}\left(k-1\right) \end{array}\right]\times {\left[\begin{array}{cc} I_{n} & 0\\ 0 & \beta_{i}\left(k\right) \end{array}\right]}^{T}+\left[\begin{array}{cc} 0 & 0\\ 0 & {\overset{¯}{\sigma }}_{\eta_{i}}^{2}\left(k\right) \end{array}\right]\text{.} \end{array}$$

Defining $\Delta \Lambda_{ii}\left(k\right)=\Lambda_{ii}\left(k\right)-{\overset{¯}{\Lambda }}_{ii}\left(k\right)$, $\Delta Q\left(k-1\right)=Q\left(k-1\right)-\overset{¯}{Q}\left(k-1\right)$, $\Delta \sigma_{\eta_{i}}^{2}\left(k\right)=\sigma_{\eta_{i}}^{2}\left(k\right)-{\overset{¯}{\sigma }}_{\eta_{i}}^{2}\left(k\right)$, it yields

(33) $$\begin{array}{c} \Delta \Lambda_{ii}\left(k\right)=\left[\begin{array}{cc} I_{n} & 0\\ 0 & \beta_{i}\left(k\right) \end{array}\right]\left[\begin{array}{cc} \Delta Q\left(k-1\right) & \Delta Q\left(k-1\right)\\ \Delta Q\left(k-1\right) & \Delta Q\left(k-1\right) \end{array}\right]\times {\left[\begin{array}{cc} I_{n} & 0\\ 0 & \beta_{i}\left(k\right) \end{array}\right]}^{T}+\left[\begin{array}{cc} 0 & 0\\ 0 & \Delta \sigma_{\eta_{i}}^{2}\left(k\right) \end{array}\right]\text{,} \end{array}$$

According to Assumption 1, we have Δ*Q*(*k−*1)*≥*0, Δ*σ*_*η*_*i*__^2^(*k*)*≥*0, and combined with Lemmas 2 and 3, it obviously yields ΔΛ_*ii*_(*k*)*≥*0.

Define

(34) $$\begin{array}{c} \Delta_{i}\left(k|k\right)=P_{i}\left(k|k\right)-{\overset{¯}{P}}_{i}\left(k|k\right)\text{.} \end{array}$$

Subtracting (23) from (22) yields the Lyapunov equation

(35) $$\begin{array}{c} \Delta_{i}\left(k\left|k\right.\right)=\Psi_{fi}\left(k\right)\Delta_{i}\left(k-1\left|k-1\right.\right)\Psi_{fi}^{T}\left(k\right)+U_{i}\left(k\right)\text{,} \end{array}$$

(36) $$\begin{array}{c} U_{i}\left(k\right)=\Gamma_{i}^{\gamma_{i}}\left(k\right)\Delta {\overset{¯}{\Lambda }}_{ii}\left(k\right){\left(\Gamma_{j}^{\gamma_{i}}\left(k\right)\right)}^{T}\text{.} \end{array}$$

At the moment *k*, *γ*_*i*_(*k*) of the *i*th sensor takes the unique value; then, Γ_*i*_^*γ*_*i*_^(*k*) in (36) is unique, too. From $\Delta {\overset{¯}{\Lambda }}_{ii}\left(k\right)\geq 0$ and *U*_*i*_(*k*)*≥*0, according to Assumption 2, $\Delta_{i}\left(0|0\right)=P_{i}\left(0|0\right)-{\overset{¯}{P}}_{i}\left(0|0\right)\geq 0$; therefore, from (35), it yields Δ_*i*_(1*|*1)*≥*0, and applying the mathematical induction for all moments *k* yields Δ_*i*_(*k|k*)*≥*0 , that is, (31) holds. In particular, if we take $\overset{¯}{Q}\left(k\right)=Q\left(k\right)$, ${\overset{¯}{\sigma }}_{\eta_{i}}^{2}\left(k\right)=\sigma_{\eta_{i}}^{2}\left(k\right)$ and ${\overset{¯}{P}}_{i}\left(0|0\right)=P_{i}\left(0|0\right)$ satisfying Assumptions 1 and 2, for any moment *k≥*0, we have *U*_*i*_(*k*)*=*0 and Δ_*i*_(0*|*0)*=*0. Therefore, applying (35) obtains Δ_*i*_(1*|*1)*=*0 and applying the mathematical induction for all moments *k* yields Δ_*i*_(*k|k*)*=*0, i.e., ${\overset{¯}{P}}_{i}\left(k|k\right)=P_{i}\left(k|k\right),k\geq 0$. For any other upper bound *P*_*i*_^*∗*^(*k|k*), it yields $P_{i}\left(k|k\right)={\overset{¯}{P}}_{i}\left(k|k\right)\leq P_{i}^{*}\left(k|k\right)$, which means that *P*_*i*_(*k|k*) is the minimal upper bound of ${\overset{¯}{P}}_{i}\left(k|k\right)$. The proof is completed.

## 4. Robust SCI Fusion Estimation Algorithm

**Figure 2:**
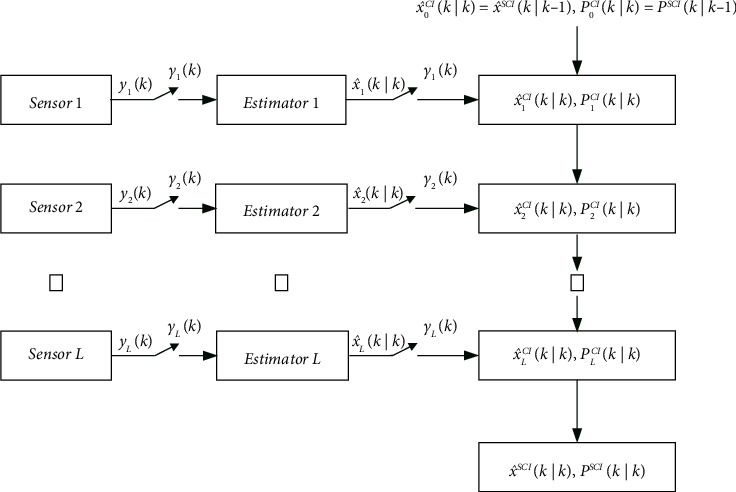
Structure block diagram of the event-triggered SCI fusion estimation algorithm.

Based on the SCI fusion estimation algorithm [24] and the adaptive innovation event-triggered mechanism, the sequential fusion processing of a networked system which meets the triggering conditions can reduce the computational burden on the fusion center and maintain good real-time performance. The structure block diagram of the event-triggered SCI fusion estimation algorithm is shown in Figure 2. The structure block diagram of the event-triggered SICI fusion estimation algorithm is similar to that of the event-triggered SCI fusion estimation algorithm, which is omitted.

**Theorem 2 .**

Based on the adaptive innovation event-triggered mechanism, the robust SCI fusion estimator for the multi-sensor networked systems (1)–(3) with known conservative upper bounds *Q*(*k*), *R*_*i*_(*k*), and *S*_*i*_(*k*) of noise variances is

(37) x^iCIk|k=1−γikx^i−1CIk|k+γikPiCIk|k×ωikPi−1CIk|k−1x^i−1CIk|k+1−ωikPik|k−1x^ik|k,

(38) $$\begin{array}{c} P_{i}^{CI}\left(k|k\right)=\left[1-\gamma_{i}\left(k\right)\right]P_{i-1}^{CI}\left(k|k\right)+\gamma_{i}\left(k\right)\{\omega_{i}\left(k\right){\left[P_{i-1}^{CI}\left(k|k\right)\right]}^{-1}+\left[1-\omega_{i}\left(k\right)\right]{\left[P_{i}\left(k|k\right)\right]}^{-1}{\}}^{-1}\text{,} \end{array}$$

(39) x^SCIk|k=x^LCIk|k,PSCIk|k=PLCIk|k,

where

(40) x^0CIk|k=x^SCIk|k−1,P0CIk|k=PSCIk|k−1,

(41) x^SCI0|−1=μ0,PSCI0|−1=P0.

The minimization performance index of the optimal weighting factor *ω*_*i*_(*k*) is

(42) $$\begin{array}{c} \min_{\omega_{i}\left(k\right)\in \left[0,1\right]}trP_{i}^{CI}\left(k|k\right)=\min_{\omega_{i}\left(k\right)\in \left[0,1\right]}tr{\left\{\omega_{i}\left(k\right){\left[P_{i-1}^{CI}\left(k|k\right)\right]}^{-1}+\left[1-\omega_{i}\left(k\right)\right]{\left[P_{i}\left(k|k\right)\right]}^{-1}\right\}}^{-1}\text{.} \end{array}$$

**Proof**

The SCI fusion prediction at the current moment is used as the estimation of the 0th sensor for CI fusion with the estimation of the 1st sensor. When *γ*_*i*_(*k*)*=*1, the *i*th fusion fuses the fusion estimation of the (*i−*1)th fusion with the estimation of the *i*th estimator for CI fusion and transmits it to the (*i+*1)th fusion. When *γ*_*i*_(*k*)*=*0, the CI fusion cannot be performed by the *i*th fusion, so at this time the fusion estimation is the (*i−*1)th fusion result and is transmitted to the (*i+*1)th fusion. Therefore, the adaptive innovation event-triggered mechanism can be added on the basis of the original SCI fusion estimation algorithm. The proof is completed.

**Corollary 1 .**

The conservative and the actual fusion filtering error variance matrices of the event-triggered SCI fusion estimation algorithm, respectively, are

(43) $$\begin{array}{c} P^{SCI}\left(k|k\right)={\left[\overset{l\left(k\right)}{\underset{Ji=1}{\sum }}\theta_{Ji}^{\left(l\left(k\right)\right)}\left(k\right)P_{Ji}^{-1}\left(k|k\right)\right]}^{-1}\text{,}\\ \overset{l\left(k\right)}{\underset{Ji=1}{\sum }}\theta_{Ji}^{\left(l\left(k\right)\right)}\left(k\right)=1,\theta_{Ji}^{\left(l\left(k\right)\right)}\left(k\right)\geq 0\text{,}\\ {\overset{¯}{P}}^{SCI}\left(k|k\right)=P^{SCI}\left(k|k\right)\left[\overset{l\left(k\right)}{\underset{Ji=1}{\sum }}\overset{l\left(k\right)}{\underset{Jj=1}{\sum }}\theta_{Ji}^{\left(l\left(k\right)\right)}\left(k\right)\theta_{Jj}^{\left(l\left(k\right)\right)}\left(k\right)\times P_{Ji}^{-1}\left(k|k\right){\overset{¯}{P}}_{JiJj}\left(k|k\right)P_{Jj}^{-1}\left(k|k\right)\right]P^{SCI}\left(k|k\right)\text{,} \end{array}$$

where *l*(*k*) is the number of the triggered sensors at moment *k* (including the 0th sensor), 1*≤l*(*k*)*≤L+*1, and renumbering these sensors, *J*_*i*_ is the serial number of the triggered sensors at moment *k*, 1*≤J*_1_*<⋯<J*_*l*(*k*)_*≤L+*1, denoting *P*_*Ji*_(*k|k*) as the conservative local filtering error variance matrix of the *J*_*i*_ sensor at moment *k* and ${\overset{¯}{P}}_{JiJj}\left(k|k\right)$ as the actual local filtering error cross-variance matrix of *J*_*i*_ sensor and *J*_*j*_ sensor at moment *k*. The weight coefficient *θ*_*Ji*_^(*r*)^ can be calculated recursively:

(44) $$\begin{array}{c} \theta_{Ji}^{\left(r\right)}\left(k\right)=\omega^{\left(r-1\right)}\left(k\right)\theta_{Ji}^{\left(r-1\right)}\left(k\right),J_{i}=1,\cdots ,r-1\text{,}\\ \theta_{r}^{\left(r\right)}\left(k\right)=1-\omega^{\left(r-1\right)}\left(k\right),r=2,\cdots ,l\left(k\right)\text{,} \end{array}$$

with initial value

(45) $$\begin{array}{c} \theta_{1}^{\left(2\right)}\left(k\right)=\omega^{\left(1\right)}\left(k\right),\theta_{2}^{\left(2\right)}\left(k\right)=1-\omega^{\left(1\right)}\left(k\right)\text{.} \end{array}$$

**Proof**

The proof is similar to Theorem 4.12 and Theorem 4.13 in [33]. Note that due to the adaptive innovation event-triggered mechanism, the number of sensors at moment *k* is not *L* but *l*(*k*). The proof is completed.

**Corollary 2 .**

Under Assumptions 1 and 2, the actual SCI fusion estimation algorithm ((37)–(41)) based on the adaptive innovation event-triggered mechanism is robust, i.e.,

(46) $$\begin{array}{c} {\overset{¯}{P}}^{SCI}\left(k|k\right)\leq P^{SCI}\left(k|k\right)\text{.} \end{array}$$

**Proof**

The conclusion of Theorem 1 is available for all *l*(*k*) sensors triggered at time *k*. Applying the mathematical induction on this basis and combining it with Theorem 4.10 in [33], the conclusion (46) can be directly introduced. The proof is completed.

## 5. Robust SICI Fusion Estimation Algorithm

It is known that CI fusion algorithm is too conservative, while ICI fusion algorithm is a new method to deal with the unknown correlation between local estimations, which is less conservative than CI fusion algorithm, so the following theorem is proposed by using ICI fusion algorithm.

**Theorem 3 .**

Based on the adaptive innovation event-triggered mechanism, the robust SICI fusion estimation algorithm for the multi-sensor networked system ((1)–(3)) with known conservative upper bounds *Q*(*k*), *R*_*i*_(*k*) and *S*_*i*_(*k*) of noise variances is

(47) x^iICIk|k=1−γikx^i−1ICIk|k+γik×KICIkx^i−1ICIk|k+LICIkx^ik|k,

(48) $$\begin{array}{c} P_{i}^{ICI}\left(k|k\right)=\left[1-\gamma_{i}\left(k\right)\right]P_{i-1}^{ICI}\left(k|k\right)+\gamma_{i}\left(k\right)\{{\left[P_{i-1}^{ICI}\left(k|k\right)\right]}^{-1}+{\left[P_{i}\left(k|k\right)\right]}^{-1}-{\left[\omega_{i}\left(k\right)P_{i-1}^{ICI}\left(k|k\right)+\left(1-\omega_{i}\left(k\right)\right)P_{i}\left(k|k\right)\right]}^{-1}{\}}^{-1}\text{,} \end{array}$$

(49) $$\begin{array}{c} K^{ICI}\left(k\right)=P_{i}^{ICI}\left(k\left|k)\{(P_{i-1}^{ICI}(k\right|k\right){)}^{-1}-\omega_{i}\left(k\right)\left.\times {\left[\omega_{i}\left(k\right)P_{i-1}^{ICI}\left(k|k\right)+\left(1-\omega_{i}\left(k\right)\right)P_{i}\left(k|k\right)\right]}^{-1}\right\}\text{,} \end{array}$$

(50) $$\begin{array}{c} L^{ICI}\left(k\right)=P_{i}^{ICI}\left(k|k\right)\{P_{i}^{-1}\left(k|k\right)-\left(1-\omega_{i}\left(k\right)\right)\left.\times {\left[\omega_{i}\left(k\right)P_{i-1}^{ICI}\left(k|k\right)+\left(1-\omega_{i}\left(k\right)\right)P_{i}\left(k|k\right)\right]}^{-1}\right\}\text{,} \end{array}$$

(51) x^SICIk|k=x^LICIk|k,PSICIk|k=PLICIk|k,

(52) x^0ICIk|k=x^SICIk|k−1,P0ICIk|k=PSICIk|k−1,

(53) x^SICI0|−1=μ0,PSICI0|−1=P0.

The minimization performance index of the optimal weighting factor *ω*_*i*_(*k*) is

(54) $$\begin{array}{c} \min_{\omega_{i}\left(k\right)\in \left[0,1\right]}trP_{i}^{ICI}\left(k|k\right)=\min_{\omega_{i}\left(k\right)\in \left[0,1\right]}tr{\left\{{\left[P_{i-1}^{ICI}\left(k|k\right)\right]}^{-1}+{\left[P_{i}\left(k|k\right)\right]}^{-1}-{\left[\omega_{i}\left(k\right)P_{i-1}^{ICI}\left(k|k\right)+\left(1-\omega_{i}\left(k\right)\right)\times P_{i}\left(k|k\right)\right]}^{-1}\right\}}^{-1}\text{.} \end{array}$$

**Proof**

The proof process is similar to that of Theorem 2 and will not be repeated here.

**Theorem 4 .**

The actual SICI fusion filtering error variance matrix based on the adaptive innovation event-triggered mechanism is

(55) $$\begin{array}{c} {\overset{¯}{P}}^{SICI}\left(k|k\right)=\overset{l\left(k\right)}{\underset{Ji=1}{\sum }}\overset{l\left(k\right)}{\underset{Jj=1}{\sum }}\theta_{Ji}\left(k\right){\overset{¯}{P}}_{JiJj}\left(k|k\right)\theta_{Jj}^{T}\left(k\right)\text{,} \end{array}$$

Also, the weight coefficient *θ*_*Ji*_^(*r*)^ can be calculated as

(56) $$\begin{array}{c} \theta_{Ji}^{\left(r\right)}\left(k\right)=K_{ICI}^{\left(r-1\right)}\left(k\right)\theta_{Ji}^{\left(r-1\right)}\left(k\right)\text{,}Ji=1,\cdots ,r-1\text{,}\\ \theta_{r}^{\left(r\right)}\left(k\right)=I_{n}-K_{ICI}^{\left(r-1\right)}\left(k\right)\text{,}r=2,\cdots ,l\left(k\right)\text{,} \end{array}$$

with the initial values

(57) $$\begin{array}{c} \theta_{1}^{\left(2\right)}\left(k\right)=K_{ICI}^{\left(1\right)}\left(k\right),\theta_{2}^{\left(2\right)}\left(k\right)=I_{n}-K_{ICI}^{\left(1\right)}\left(k\right)\text{.} \end{array}$$

**Proof**

By [23], when *l*(*k*) sensors are triggered at *k* moment, based on the adaptive innovation event-triggered mechanism, the SICI fusion estimation algorithm can be rewritten in the form of a batch process:

(58) x^SICIk|k=∑JilkθJirkx^Jik|k,∑JilkθJirk=In.

Also, the weight coefficient *θ*_*Ji*_^(*r*)^ can be calculated as

(59) $$\begin{array}{c} \theta_{Ji}^{\left(r\right)}\left(k\right)={K_{ICI}^{\left(r-1\right.}}^{)}\left(k\right)\theta_{Ji}^{\left(r-1\right)}\left(k\right)\text{,}Ji=1,\cdots ,r-1\text{,}\\ \theta_{r}^{\left(r\right)}\left(k\right)=I_{n}-K_{ICI}^{\left(r-1\right)}\left(k\right)\text{,}r=2,\cdots ,l\left(k\right)\text{,} \end{array}$$

with the initial values

(60) $$\begin{array}{c} \theta_{1}^{\left(1\right)}\left(k\right)=K_{ICI}^{\left(1\right)}\left(k\right),\theta_{2}^{\left(2\right)}\left(k\right)=I_{n}-K_{ICI}^{\left(1\right)}\left(k\right)\text{.} \end{array}$$

The actual filtering error is

(61) x˜SICIk|k=xk−x^SICIk|k=∑JilkθJirkx˜Jik|k.

Therefore, the actual fusion error variance matrix is

(62) $$\begin{array}{c} {\overset{¯}{P}}^{SICI}\left(k|k\right)=E\left[{\overset{˜}{x}}^{SICI}\left(k\left|k)({\overset{˜}{x}}^{SICI}(k\right|k\right){)}^{T}\right]=\overset{l\left(k\right)}{\underset{Ji=1}{\sum }}\overset{l\left(k\right)}{\underset{Jj=1}{\sum }}\theta_{Ji}\left(k\right){\overset{¯}{P}}_{JiJj}\left(k|k\right)\theta_{Jj}^{T}\left(k\right)\text{.} \end{array}$$

The proof is completed.

**Remark 2 .**

Unlike the event-triggered SCI fusion estimation algorithm, Theorem 4 does not require the use of the robust fusion filtering error variance matrix *P*^*SICI*^(*k|k*) in the computation of ${\overset{¯}{P}}^{SICI}\left(k|k\right)$. Therefore, the batch expression form of ${\overset{¯}{P}}^{SICI}\left(k|k\right)$ is not given.

**Theorem 5 .**

Under Assumptions 1 and 2, the actual SICI fusion estimation algorithm ((47)–(53)) based on the adaptive innovation event-triggered mechanism is robust, i.e.,

(63) $$\begin{array}{c} {\overset{¯}{P}}^{SICI}\left(k|k\right)\leq P^{SICI}\left(k|k\right)\text{.} \end{array}$$

**Proof**

According to [20], if the local estimation to be fused is robust, i.e., ${\overset{¯}{P}}_{i}\left(k|k\right)\leq P_{i}\left(k|k\right)$, the two-sensor ICI fusion is also robust, i.e., ${\overset{¯}{P}}^{ICI}\left(k|k\right)\leq$*P*^*ICI*^(*k|k*). For the actual event-triggered SICI fusion estimation algorithm, *l*(*k*) sensors triggered at the moment *k* have the conclusions of Theorem 1, i.e., ${\overset{¯}{P}}_{Ji}\left(k|k\right)\leq P_{Ji}\left(k|k\right)$. The robustness of the two-sensor ICI fusion can be induced by the mathematical induction as ${\overset{¯}{P}}_{Ji}^{ICI}\left(k|k\right)\leq P_{Ji}^{ICI}\left(k|k\right)$, *J*_*i*_*=*1,*⋯*,*l*(*k*). In particular, there exists that ${\overset{¯}{P}}_{l\left(k\right)-1}^{ICI}\left(k|k\right)\leq P_{l\left(k\right)-1}^{ICI}\left(k|k\right)$, and according to the structure of the robust SICI fusion estimation algorithm based on the event-triggered mechanism, it yields *P*_*l*(*k*)*−*1_^*ICI*^(*k|k*)*=P*_*L*_^*ICI*^(*k|k*)*=P*^*SICI*^(*k|k*), and ${\overset{¯}{P}}_{l\left(k\right)-1}^{ICI}\left(k|k\right)={\overset{¯}{P}}^{SICI}\left(k|k\right)$, so ${\overset{¯}{P}}^{SICI}\left(k|k\right)\leq$*P*^*SICI*^(*k|k*). The proof is completed.

**Remark 3 .**

Compared with the SCI fusion algorithm, the SICI fusion algorithm is computationally intensive due to its multiple inverse operations, but it has higher accuracy, better tightness, and better consistency [20]. Therefore, the robust event-triggered SICI fusion algorithm has similar properties.

## 6. Simulation Example

Consider a three-sensor target tracking system with uncertain noise variances and correlated noises:

(64) $$\begin{array}{c} x\left(k+1\right)=\Phi x\left(k\right)+\Gamma w\left(k\right)\text{,}\\ \Phi =\left[\begin{array}{ccc} 1 & T & \frac{T^{2}}{2}\\ 0 & 1 & T\\ 0 & 0 & 1 \end{array}\right]\text{,}\\ \Gamma =\left[\begin{array}{c} \frac{T^{2}}{2}\\ T\\ 1 \end{array}\right]\text{,}\\ y_{i}\left(k\right)=H_{i}x\left(k\right)+v_{i}\left(k\right)\text{,}\\ v_{i}\left(k\right)=\eta_{i}\left(k\right)+\beta_{i}w\left(k-1\right),i=1,2,3\text{,} \end{array}$$

where the sampling time *T=*0.01, the observation matrices *H*_1_*=*[1,0,0], *H*_2_*=*[0,1,0], and *H*_3_*=*[0,0,1], and *w*(*k*) and *η*_*i*_(*k*) are uncorrelated white noises with zero mean and conservative upper bounds of noise variances *σ*_*w*_^2^ and *σ*_*η*_*i*__^2^, respectively.

In simulation, we take *σ*_*w*_^2^*=*0.16, *σ*_*η*_1__^2^*=*0.64, *σ*_*η*_2__^2^*=*0.36, *σ*_*η*_3__^2^*=*0.49, *β*_1_*=*0.2, *β*_2_*=*0.4, *β*_3_*=*0.1, and each conservative upper bound of noise variances *Q=*Γ*σ*_*w*_^2^Γ^T^, *R*_*ij*_*=β*_*i*_*σ*_*w*_^2^*β*_*j*_*+σ*_*η*_*i*__^2^*δ*_*ij*_, *δ*_*ii*_*=*1, *δ*_*ij*_*=*0(*i≠j*), *S*_*i*_*=*Γ*σ*_*w*_^2^*β*_*i*_. Take the initial value of state as *x*(0)*=*[0.3,0.1,0.2]^T^ and the initial value of error variance as *P*(0)*=I*_3_. The initial values of each state estimates are x^i0|−1=x^SCI0|−1=x^SICI0|−1=0.3,0.1,0.2T, and the initial values of the error variances are *P*_*i*_(0*|−*1)*=P*^*SCI*^(0*|−*1)*=P*^*SICI*^(0*|−*1)*=I*_3_. Set the parameters of the adaptive threshold as *a=*0.6, *b=*0.2, *c=*0.9.

**Figure 3:**
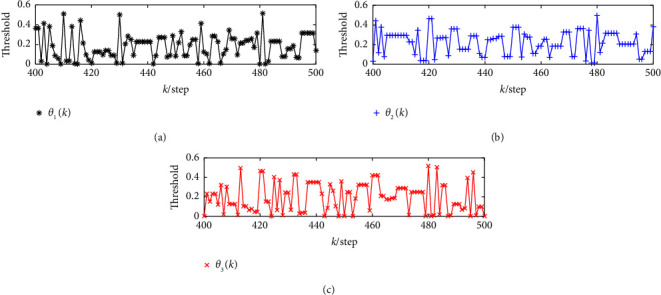
Threshold change of adaptive innovation event-triggered mechanism. (a) Threshold change for the first sensor. (b) Threshold change for the second sensor. (c) Threshold change for the third sensor.

In order to clearly demonstrate the adaptive influence of the adaptive innovation event-triggered mechanism threshold, the thresholds of three sensor changes at the step *k=*300*−*400 are selected here, as shown in Figure 3. It can be seen that the thresholds will adaptively adjust according to the changes of the innovation, where the thresholds will produce a change when the sensor is not triggered at that moment. Moreover, the communication rates of the three sensors are calculated to be 60.67%, 48.5%, and 70.67%, respectively. The effectiveness of the presented mechanism is confirmed.

**Figure 4:**
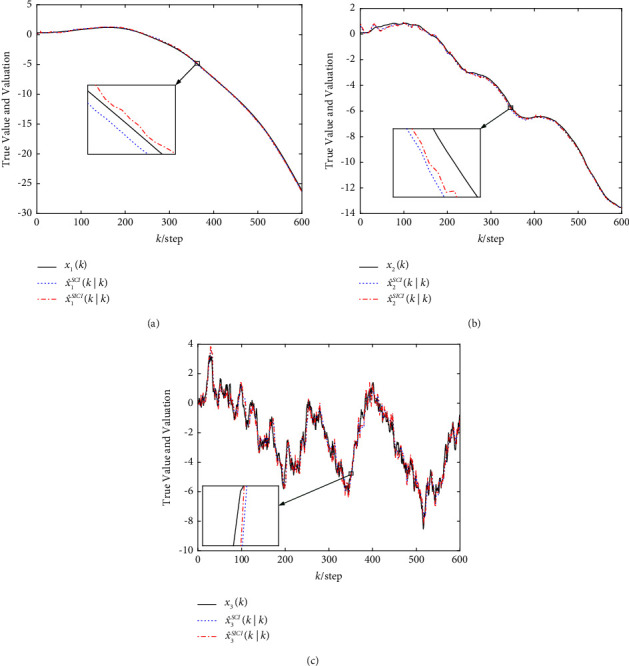
True value and fusion estimation curves. (a) True value and fusion estimation of the location *x*_1_(*k*). (b) True value and fusion estimation of the speed *x*_2_(*k*). (c) True value and fusion estimation of the acceleration *x*_3_(*k*).

The simulation results of the robust SCI and SICI fusion estimation algorithms based on the adaptive innovation event-triggered mechanism are shown in Figure 4, which shows that both robust event-triggered fusion estimation algorithms track well and have effectiveness.

**Figure 5:**
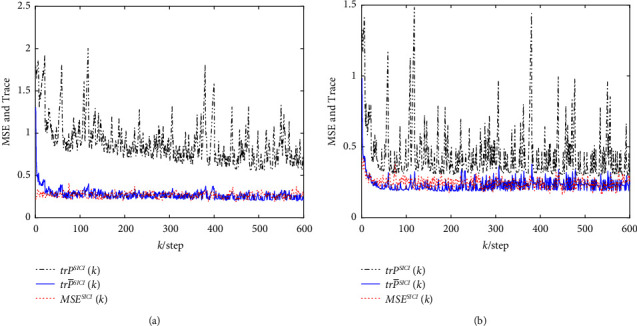
The MSE and the error variance trace curves of two robust event-triggered fusion estimation algorithms. (a) The MSE and the error variance trace curves of the robust event-triggered SCI fusion estimation algorithm. (b)The MSE and the error variance trace curves of the robust event-triggered SICI fusion estimation algorithm.

The mean square error (MSE) and the error variance curves of the robust event-triggered SCI and SICI fusion estimation algorithms are shown in Figure 5 for 100 Monte Carlo tests, respectively, and it can be seen that their MSE values are close to the traces of the actual filtering error variances and lie below the traces of the conservative filtering error variances, which verifies the consistencies of the fused filtering error variances.

**Figure 6:**
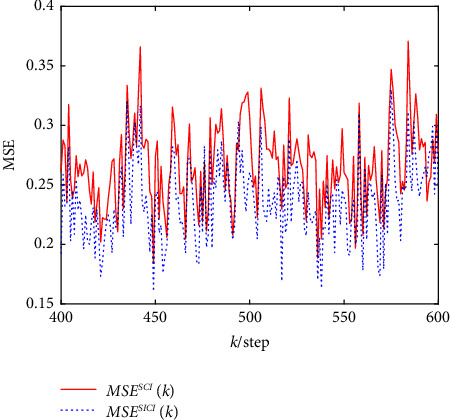
MSE of two robust event-triggered fusion estimation algorithms.

The comparison results of 100 Monte Carlo simulations formed from robust event-triggered SCI and SICI fusion estimation algorithms at the step *k=*400*−*600 are plotted in Figure 6, which shows that the robust event-triggered SICI fusion estimation algorithm is more accurate than the robust event-triggered SCI fusion estimation algorithm, and they are consistent as mentioned above.

To validate the influences of different actual noise variances satisfying Assumption 1, we select three sets of noise variances as

1. $\overset{¯}{Q}=0.8Q,{\overset{¯}{R}}_{ij}=0.8R_{ij},{\overset{¯}{S}}_{i}=0.8{\overset{¯}{S}}_{i}$
2. $\overset{¯}{Q}=0.6Q,{\overset{¯}{R}}_{ij}=0.6R_{ij},{\overset{¯}{S}}_{i}=0.6{\overset{¯}{S}}_{i}$
3. $\overset{¯}{Q}=0.4Q,{\overset{¯}{R}}_{ij}=0.4R_{ij},{\overset{¯}{S}}_{i}=0.4{\overset{¯}{S}}_{i}$

**Figure 7:**
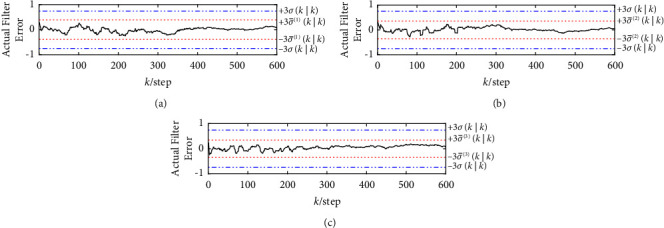
The three sets of the actual filtering errors and the corresponding robust and actual ±3-standard deviation bounds for the robust event-triggered SCI fusion estimation algorithm. (a) The actual filtering errors and the corresponding robust and actual ±3-standard deviation bounds of the first group. (b) The actual filtering errors and the corresponding robust and actual ±3-standard deviation bounds of the second group. (c) The actual filtering errors and the corresponding robust and actual ±3-standard deviation bounds of the third group.

**Figure 8:**
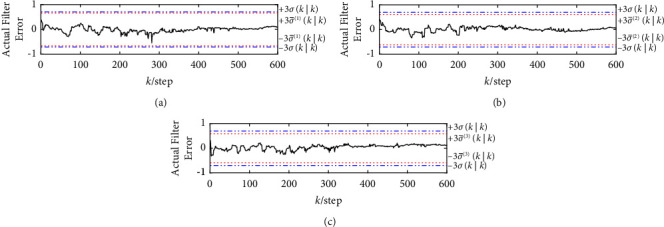
The three sets of the actual filtering errors and the corresponding robust and actual ±3-standard deviation bounds for the robust event-triggered SICI fusion estimation algorithm. (a) The actual filtering errors and the corresponding robust and actual ±3-standard deviation bounds of the first group. (b) The actual filtering errors and the corresponding robust and actual ±3-standard deviation bounds of the second group. (c) The actual filtering errors and the corresponding robust and actual ±3-standard deviation bounds of the third group.

The curves of three sets of the actual filtering errors, the robust and actual standard deviation bounds ±3*σ*(*k|k*) and $\pm 3{\overset{¯}{\sigma }}^{\left(m\right)}\left(k|k\right)\left(m=1,2,3\right)$ are shown in Figures 7 and 8, where the solid curve indicates the actual fusion filtering errors, and the dashed and dotted lines indicate the robust and actual ±3-standard deviation bounds, respectively. It can be seen that more than 99% of the actual fusion filtering error values of two robust event-triggered fusion estimation algorithms lie between $\pm 3{\overset{¯}{\sigma }}^{\left(m\right)}\left(k|k\right)$, and all three groups have the relation $3{\overset{¯}{\sigma }}^{\left(m\right)}\left(k|k\right)\leq 3\sigma \left(k|k\right)$, which verifies the correctness of ${\overset{¯}{P}}^{SCI}\left(k|k\right)$, ${\overset{¯}{P}}^{SICI}\left(k|k\right)$ and the robustness of two robust event-triggered fusion estimation algorithms.

**Figure 9:**
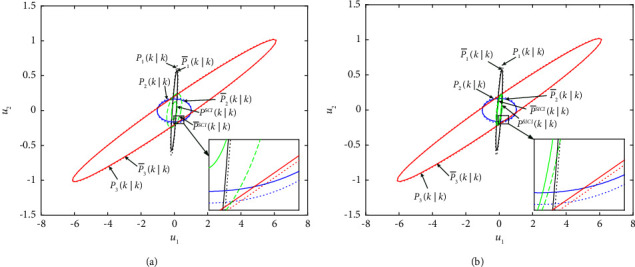
Conservative and real covariance ellipses. (a) Conservative and real covariance ellipses for the robust event-triggered SCI fusion estimation algorithm. (b) Conservative and real covariance ellipses for the robust event-triggered SICI fusion estimation algorithm.

The conservative and actual error covariance ellipses of robust event-triggered SCI and SICI fusion estimation algorithms are shown in Figure 9, where the local conservative error covariance ellipses of each sensor contain the local actual error covariance ellipses, and three local conservative error covariance ellipses contain the conservative error covariance ellipses of two robust event-triggered estimation fusion algorithms. It also illustrates that the robust accuracies of two robust event-triggered fusion estimation algorithms are higher than those of each sensor, which verifies the robustness and effectiveness of the robust local filtering estimation algorithm and two robust event-triggered fusion estimation algorithms.

## 7. Conclusion

An adaptive innovation event-triggered mechanism is designed in this paper, which can adaptively adjust the threshold value according to the innovation and reduce the communication burden of the networked system. Under this mechanism, the robust local filter is proposed for the uncertain networked systems with correlated noises, and its robustness is demonstrated under different triggered cases using the Lyapunov equation. In order to avoid the calculation of the cross-covariances in multi-sensor networked systems, two robust event-triggered sequential fusion estimation algorithms are proposed using SCI and SICI fusion ideas, respectively, and their actual fusion error variances are obtained by converting the two robust fusion estimation algorithms into a batch form, whose robustness is proved under the adaptive innovation event-triggered mechanism. The simulation example illustrates that the proposed robust event-triggered sequential fusion estimation algorithms work well with the unknown actual noise variances, and they have robustness in the case of only knowing the noise variance upper bounds and can reduce communication rate and noise correlations.

## Data Availability

The data that support the findings of this study are available from the corresponding author upon reasonable request.
